# Bioinspired 5-caffeoylquinic acid capped silver nanoparticles using *Coffee arabica* leaf extract for high-sensitive cysteine detection

**DOI:** 10.1038/s41598-023-34944-9

**Published:** 2023-05-27

**Authors:** E. S. Harsha Haridas, Susmita Bhattacharya, M. K. Ravi Varma, Goutam Kumar Chandra

**Affiliations:** 1 Department of Physics, National Institute of Technology, Kozhikode, Kerala 673601 India; 2 Radiation Laboratory, University of Notre Dame, Notre Dame, IN 46556 USA

**Keywords:** Biosynthesis, Green chemistry, Nanoparticles, Bioinspired materials

## Abstract

Selection of plant extracts as bioactive phytochemical source to synthesize nanoparticles is highly demanding due to the biocompatibility, nontoxicity, and cost-effectiveness over other available physical and chemical methods. Here, for the first time, *Coffee arabica* leaf extracts (CAE) were used to produce highly stable silver nanoparticles (AgNPs) and the corresponding bio reduction, capping and stabilization mechanism mediated by dominant isomer 5-caffeoylquinic acid (5-CQA) is discussed. UV–Vis, FTIR, μRaman spectroscopy, TEM, DLS and Zeta potential analyzer measurements were employed to characterize these green synthesized NPs. The affinity of 5-CQA capped CAE–AgNPs to thiol moiety of amino acid is utilized for the selective as well as sensitive detection of L-cysteine (L-Cys) to a low detection limit of 0.1 nM, as obtained from its μRaman spectra. Hence, the proposed novel, simple, eco-friendly, and economically sustainable method can provide a promising nanoplatform in the field of biosensors compliant with large-scale industrial production of AgNPs without aid of further instrumentation.

## Introduction

The usage of silver nanoparticles (AgNPs) for various consumer goods like antibiotics, sensing devices, cosmetics, food, and engineered devices is constantly increasing, owing to their unique physical and biological properties^1–3^. Synthesis of stable AgNPs with proper distribution and properties as well as biocompatibility is important and efficient for their activities. Various methods have been reported for producing AgNPs, including chemical reduction, evaporation, laser ablation, condensation, the irradiance of laser, microwave or electrons, ionization, and photochemical routes and green synthesis using either microorganisms or various plant parts^4–7^. Except for those green synthesis routes, most existing methods use toxic chemicals as reducing and capping agents and pose environmental problems. Some are time-consuming and require controlled conditions, complicated procedures, experienced technicians, proper hygiene, and heavy investments.

Green nanotechnology is a bridge between nanotechnology and nature which inspires us to design, develop and implement green alternatives for the synthesis of nanomaterials instead of synthetic and chemical routes by eliminating harsh materials and further helps us to build sustainable future^8–10^. The contribution from green nanotechnology includes the production of safe, non-toxic, cost-effective- biosensors, biocompatible NPs, drug delivery agents, developing energy storage devices and therapeutic agents, etc.^11^. There have been many efforts made for plant-based synthesis methods (phytosynthesis) for fabricating NPs and each work differently with respect to the chemistry of major phytochemical present in the plant extract^5,7,9,10^. Since all the plant bodies are ideally biofactories, it will be easeful to synthesize functional nanomaterials from any plant extract. But the important matter under consideration is to maintain a suitable physicochemical environment for the active participation of such phytochemicals. For example, while using ascorbic acid as a reducing agent for preparing AgNPs, the polydispersity of the nanocolloids are affected, so enough care should be needed for controlling the pH of working condition^5^. These situations create complications for researchers who are focusing on a biological application even though the phytochemicals present in plant are potent antioxidant in nature. Hence, keen attention is required during the procedure of green synthesis of NPs while choosing a plant/phytochemical.

Coffee has such an avoidable significance in the common people life, a sip of coffee actuates our day. *Coffee arabica* is the most and best popular coffee type consumed all over the world. *Coffee arabica* has enrinched with presence of chlorogenic acids (CGA), caffeic acids (CA), caffeine, trigonelline, mangiferin, carbohydrates, amino acids and rutins^12,13^. According to Acidri et al. (2020), coffee leaves are the second most important part of coffee plants that possess high phenolic quantity and anti-oxidant capacity. There exist more than fifty patents related to the application of coffee leaves in industrial as well as pharmacological fields^12^. 5-caffeoylquinic acid (5-CQA) is one of the major CGA component of CAE. Compared to beans, which are already in use for NPs production the leaves of coffee are also treasure of phytochemicals^12,14–17^. But most of the cases leaves were considered low-or no-value products. To our best knowledge, there is no study reported using *Coffee arabica* leaf extracts (CAE) to synthesize AgNPs. So, it would be interesting to synthesize CAE-reduced AgNPs (CAE–AgNPs) to produce value-added products and will be economical support for coffee farmers.

Being simple, safe and cost-effective the phyto-reduced AgNPs are used as a better candidate in various molecular diagnostic processes^18,19^. The sensing ability of AgNPs is enhanced via surface plasmon resonance (SPR), spectrophotometric emission and absorption. Since AgNPs show a high affinity towards nitrogen and sulfur-containing molecules^20^, their biological and physicochemical activities can be enhanced by the functionalization of amino acids which contain the thiol and amine groups. This is one of the reasons for the increasing demand for developing AgNPs-based biosensors for L-Cys which plays a crucial role to track biological pathways. L-Cys, a non-essential amino acids, is polar and uncharged and essential for cellular systems in living things. It controls detoxification, helps in protein metabolism, and suppresses the ageing of the skin by preserving its texture and elasticity by producing collagen. The imbalance in the levels of L-Cys in biofluids induces health disorders like cystinuria, hair loss, and growth retardation, fat loss, liver damage, white blood cell loss, etc.^21–23^. Hence, it is vital to know the interactive mechanisms between L-Cys and AgNPs for better understanding of reactivity and pre-diagnosing disorders.

Even though various techniques are adopted for the sensing of L-Cys^24–30^ the requirement of specific chromophores for specific analytes, various analytes pre-treatment steps, highly expensive instruments, high energy consumption and large number of synthetic solvents limits the applicability. On the other hand, some methods of L-Cys detection suffer disadvantages due to long reaction time, limits in detection, involvement of highly sophisticated machinery, and technicians as well as suitable physical conditions and high investment. So, colorimetric detection of an analyte using green reduced AgNPs which is simple, environmentally friendly, rapid, and cost-effective is desirable to practice.

Here in, we report a new vision of production of AgNPs using natural source CAE for the first time and explore the practical value of CAE–AgNPs, in the field of amino acid sensing; specifically, L-Cys.

## Experimental

### Plant materials

Fresh and young *Coffee arabica* leaves were collected from the local coffee plantation of Vythiri village, Kerala, India. The collection of the plant material and the research work associated with this complies with relevant institutional, national, and international guidelines and legislation.

### Chemicals

Silver nitrate (AgNO_3_) with a 98% purity and L-Cys were purchased from Sigma-Aldrich Chemical Co. L-Alanine (Ala), L-Arginine (Arg), Butyric acid (Ba), L-Glutamic acid (Glu), Glycine (Gly), L-Isoleucine (Ile), L-Tryptophan (Trp) and L-Valine (Val) were purchased from Loba Chemie Pvt. Ltd. All chemicals were used without any further purification. All experiments were carried out using deionized (DI) water.

### Preparation of CAE

The leaves were washed several times with DI water to remove the dust. 10 gm of washed coffee leaves were cut into small pieces and boiled with 250 ml of DI water at 100 °C for 5 min. The extract was allowed to cool up to room temperature. The obtained yellow color extract was filtered using Whatman No.1 filter paper and the supernatant was stored at 4 °C for further studies.

### Preparation of CAE–AgNPs

**Figure 1:**
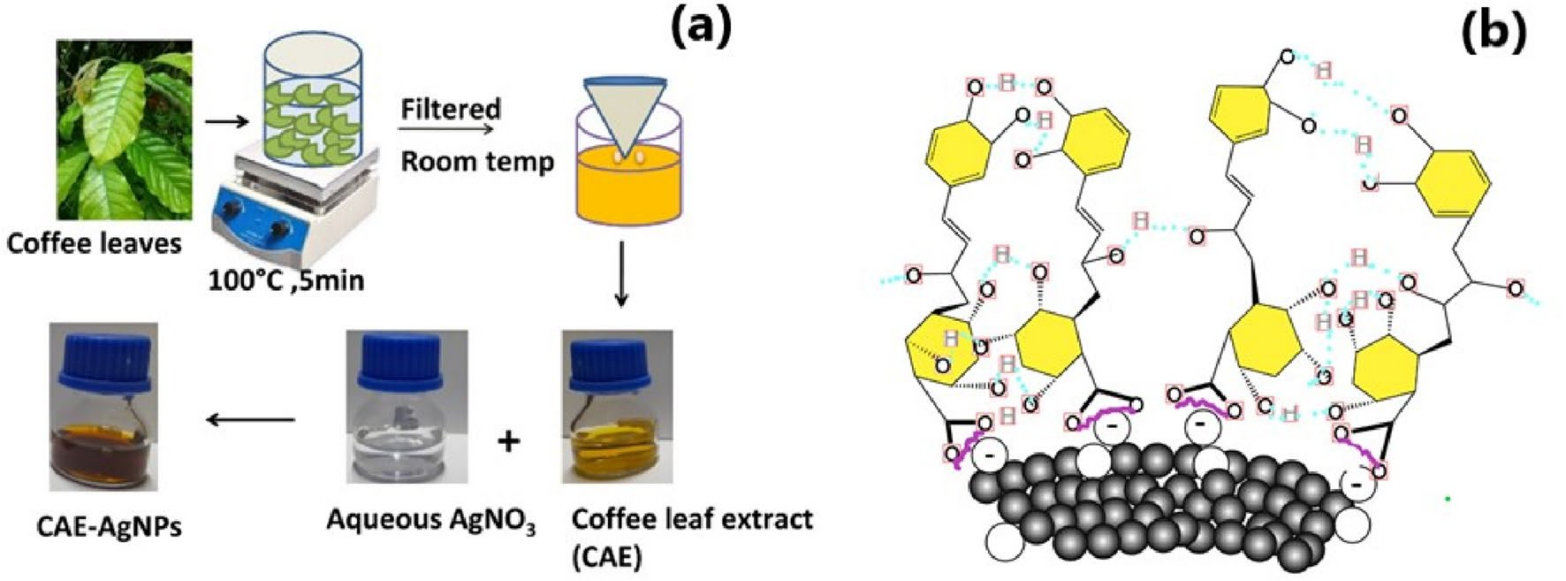
(**a**) Synthesis procedure and (**b**) mechanism of formation of CAE–AgNPs (COO^–^ groups of 5-CQA interacting with AgNPs surface via electrostatic interaction and network formed by hydrogen bonding with surface bounded molecules).

Figure 1a–b represent the schematic of the general procedure followed for the synthesis and the mechanism of formation of CAE–AgNPs, respectively. The colloidal solution of CAE–AgNPs was prepared by adding 1 ml of CAE to 5 ml aqueous solution of AgNO_3_ (1 mM) followed by gentle shaking at room temperature. Within 5–10 min the color of the solution changed from pale yellow to brownish-yellow, which indicates the reduction of Ag^+^ to Ag^0^ and the formation of CAE–AgNPs. The CAE–AgNPs suspension was also collected for different AgNO_3_ concentrations (1, 2, 3 and 4 mM) and CAE volumes (2, 3, 4 and 5 ml), respectively. The nanocolloids of green synthesized CAE–AgNPs with appropriate composition were washed by centrifugation at 14,500 rpm for 10 min. After removing the supernatant, the precipitate was redispersed in distilled water and recentrifuged. A similar cleaning procedure was repeated five times. The purified precipitate of CAE–AgNPs were dried at 60 °C for 2 h in a vacuum oven. The black-coloured residues formed were ground into powder by Hargett mortar and pestle, and then stored in an airtight container. At the stage of further analysis, these powdered CAE–AgNPs were redispersed in distilled water and used accordingly.

### Characterization

The characterization of green synthesized CAE–AgNPs was done using a UV-1800 Shimadzu double beam spectrophotometer of 1 nm resolution in the wavelength range 200–800 nm. A quartz cuvette of path length 1 cm was used for collecting the absorption spectrum. The bio-reduction of Ag^+^ in aqueous plant extract at various concentrations of CAE and metal ions was monitored by diluting the suspension using DI water. The size, morphology, and crystalline nature of the sample A1 (CAE–AgNPs suspension with 1 ml of CAE) before and after adding an adequate amount of L-Cys was investigated by high-resolution transmission electron microscopy (HR–TEM). TEM micrographs were taken using FEI Tecnai G2 Spirit Bio-Twin TEM at an accelerating voltage of 300 kV and an ultra-high-resolution pole piece. The samples were prepared by placing a drop on a graphite grid and drying it in a vacuum. Surface analysis of CAE and CAE–AgNPs was done using a PerkinElmer frontier FTIR spectrometer of Attenuated total reflection mode (FTIR–ATR) and Confocal Raman Microscope with 785 nm laser (Horiba France SAS Lab RAM HR Evolution). A particle size analyzer of Malvern Nano ZS (4 mW, 633 nm) was used for measuring surface load and the average size of A1. Stability studies were done by checking the zeta potential of AgNPs prepared using different volumes (1, 2, 3, 4, and 5 ml respectively) of CAE by Zeta Check-Particle Charge Reader (Microtrac, PMX 500).

### Selective detection of L-Cys

For the selectivity study, 1 ml of each of the nine different amino acids (Ala, Ba, Gly, Cys, Arg, Trp, Gla, Va and Isl) with 1 mM concentration were added to A1 and the color change and absorbance were noticed under the same conditions as that of the sensitivity study using UV–Vis measurements.

### Sensitive detection of L-Cys

A1 was diluted 50 times and the pH was adjusted to 6.0. 1 ml of L-Cys with different concentrations (2 × 10^–3^ to 2 × 10^–8^ M) was added to 1 ml of A1. After 5 min the color change was observed and the corresponding SPR bands were studied by UV–Vis measurements. To find out the limit of detection (LOD) using Raman measurements, additionally, two more diluted L-Cys samples were used.

## Results and discussions

### UV–Vis measurement study

**Figure 2:**
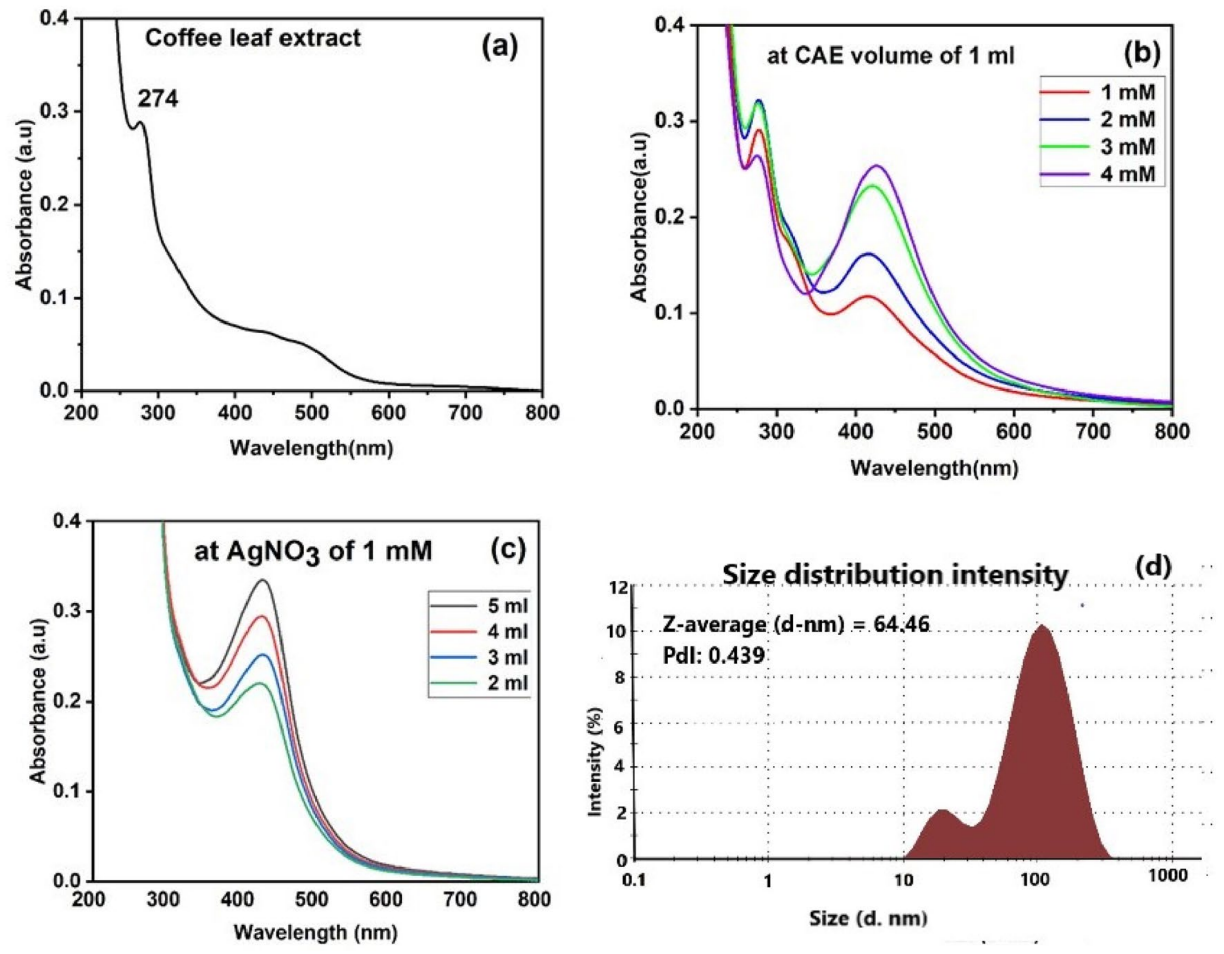
(**a**) UV-Vis spectrum of the aqueous solution of CAE. (**b**) UV-Vis spectra of CAE-AgNPs formed using various AgNO_3_ concentrations. (**c**) UV-Vis spectra of CAE-AgNPs formed using various CAE volumes. (**d**) DLS spectrum of CAE-AgNPs.

Figure 2a represents UV–Vis absorption spectrum of CAE in aqueous solution. Generally, noble metal NPs like AgNPs exhibit SPR^2^, a collective oscillation of the conduction electrons on the metal surface when they are excited by light of proper wavelengths. We have studied the NP formation by varying concentrations of metal precursor and CAE, keeping one of them at a fixed concentration (Fig. 2b,C). The optical absorption spectra of as prepared AgNPs for different concentrations of metal precursor and CAE are shown in Fig. 2b,c respectively. Figure 2d represents the hydrodynamic diameter of as prepared NP using dynamic light scattering measurements and the diameter obtained is ~ 64.46 nm. Since the redox potential of 5-CQA and Ag^+^ are found to be 0.4 and 0.8 V, respectively, therefore the reduction of Ag^+^ is thermodynamically favorable at room temperature^31–33^. From the Fig. 2b – c, it is clear that the plasmonic band position of AgNPs depends on molar ratio of CAE to Ag^+^ ions. A slightly blue shift is observed in the absorption peak when the volume of CAE varies from 2 to 5 ml indicating the reduction in particle size^18,34^. In addition, a decrease in the full-width at half maxima (FWHM) of the absorption peak occurs as the volume of the reducing agent is increased. This may be due to the donation of electron density from the surface of AgNPs to CAE^7^. These results indicate that the synthesized AgNPs using 1 ml of CAE at 3 mM AgNO_3_ concentration are the finest.

Further, we have studied the stability of this CAE–AgNPs by zeta potential measurements and it is found around − 57 mV even after three months of synthesis. The high value of zeta potential indicates the excellent stability of as-prepared samples. Thus, these CAE–AgNPs were selected for our further analysis.

**Figure 3:**
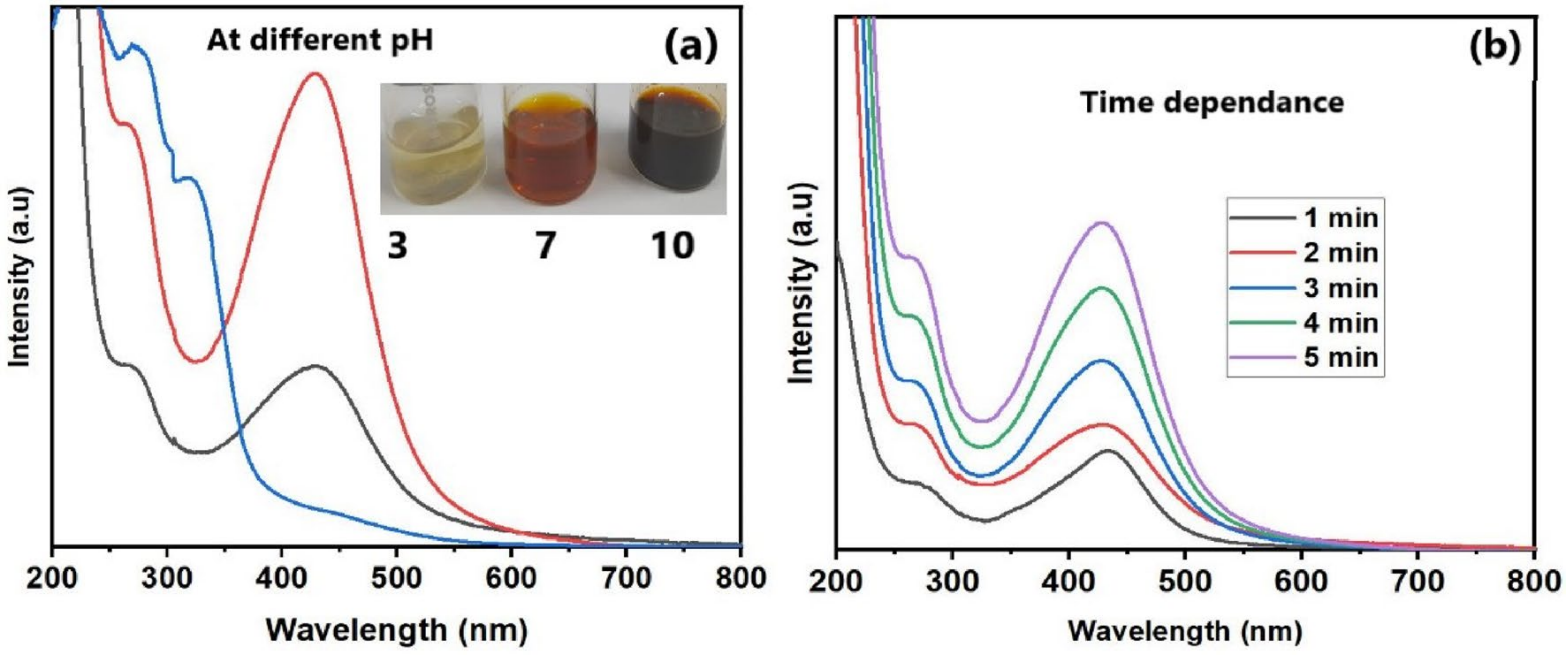
(**a**) UV-Vis spectra of CAE-AgNPs synthesized at various pH conditions (inset depicts the color of the solutions at three different pH values). (**b**) UV-Vis spectra were monitored for the initial five minutes after mixing CAE and AgNO3 solution at room temperature.

Reaction conditions especially pH of the solvent have an important role in the character of green synthesized NPs^35,36^. The synthesis of CAE–AgNPs at acidic pH (pH = 3) results in an immediate precipitate (white color) formation of suspension (inset of Fig. 3a). The UV-absorption spectrum corresponding to acidic pH indicates the failure of CAE–AgNPs formation at this condition. At neutral pH (pH = 7) a quick reaction results in the formation brown color solution. The single SPR band position at around 425 nm confirms the formation of CAE–AgNPs at this reaction condition. In contrast to acidic conditions, at basic pH (pH = 10) the formation of CAE–AgNPs occurs in a rapid manner and the solution is turned into dark brown color. An aggregation of the CAE–AgNPs is observed and the solution gets agglomerated within minutes.

The time dependence for the formation of CAE–AgNPs at neutral pH has also been investigated immediately after mixing CAE and precursor solution at room temperature (shown in Fig. 3b) to check its sustainability over time. It has been seen that growth reaction takes place in a rapid manner. Within five minutes a symmetrical SPR band with high absorbance corresponding to the characteristics of CAE–AgNPs at 425 nm is obtained and there is no notable change in SPR within 24 h. As our main focus is to synthesize biocompatible CAE–AgNPs with long-term stability, the neutral pH condition is more favourable without any drastic modification in the physicochemical characteristics. Moreover, neutral pH is suitable for in-situ synthesis and biological applications.

### Mechanism of NP formation & morphological study

**Figure 4:**
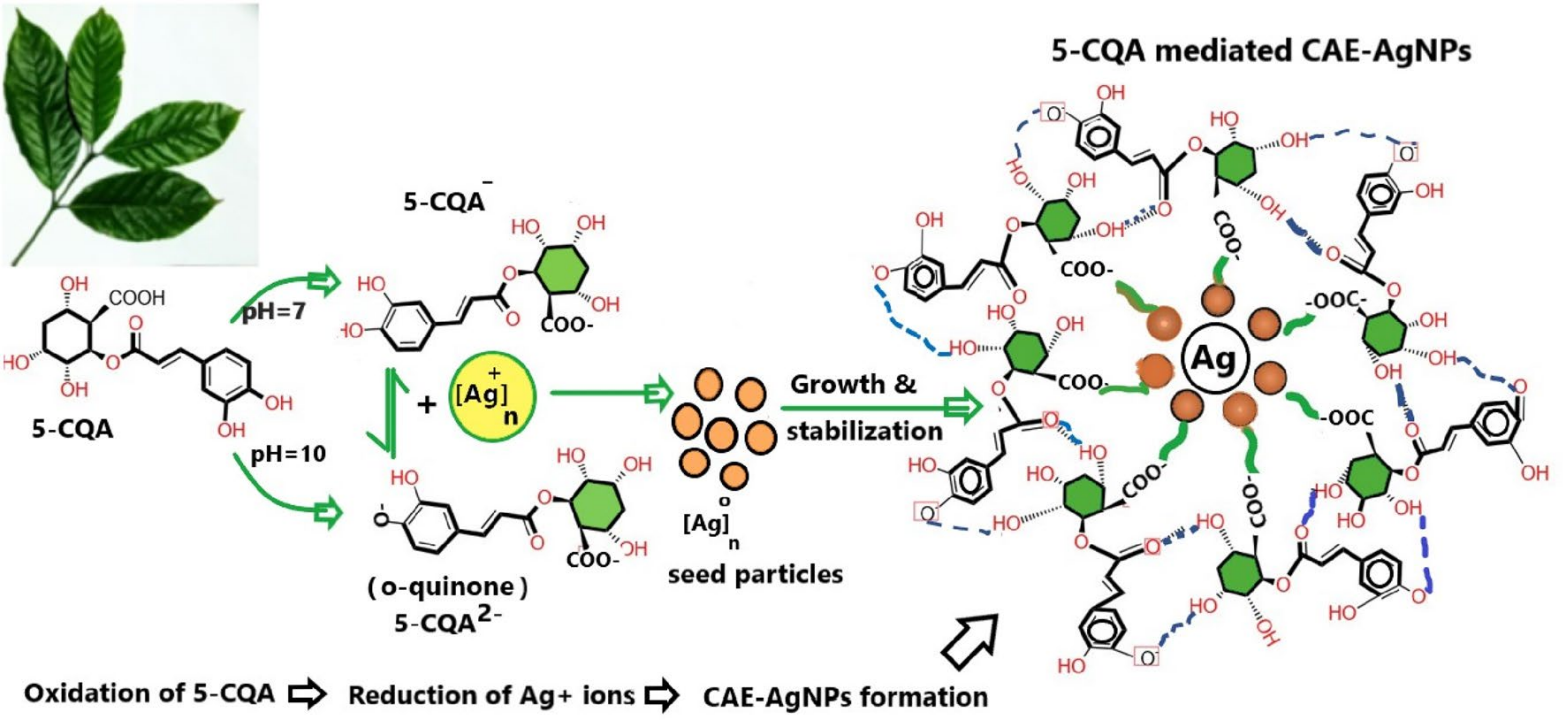
Proposed detailed mechanism of CAE-AgNPs formation mediated by 5-CQA.

Figure 4 represents the proposed detail mechanism of formation of CAE–AgNPs. A recent study by Cangeloni et al. shows a quantitative estimation of 5-CQA, caffeine, mangiferin and trigonelline, the four major phytochemicals present in CAE with a mass content in the order of 16.27 ± 1.66, 7.94 ± 0.42, 4.47 ± 0.13, 4.43 ± 0.14 g/Kg DW of coffee leaves^13^. As the number of hydroxyls group is an important factor which determines the antioxidant activity of a molecule, the reduction mechanism of Ag^+^ ions to Ag^0^ can be controlled by the dominant bioactive molecule having a higher number of hydroxyl groups in it.

The most abundant phytochemicals in CAE; CGAs are esters formed with quinic acid and trans-cinnamic acids like caffeic acid and ferulic acid in which the hydroxyl part from phenolic acid donates a hydrogen atom to free radicals that accounts for its strong hydroxyl and superoxide scavenging action^37^. As it contains larger number of hydroxyl groups, its antioxidant potential is higher than the remaining bioactive elements which has been proven already. Additionally, the biomolecular constant for scavenging hydroxyl radicals of CGA is found to be 7.73 × 10^9^ M^−1^ s^−1^, is higher than that of purine alkaloid caffeine (5.9 × 10^9^ M^−1^ s^−1^) which is the second abundant bioactive component present in CAE^12^. Therefore, we infer that 5-CQA, a major (quantitatively larger in amount) bioactive element with excellent antioxidant potential controls and mediates the formation mechanism of CAE–AgNPs. The reducing property of 5-CQA molecule depends on pH condition, structure and functional group present on it; mainly the electron-withdrawing effect of COO^−^ group (carboxyl group) on the cyclohexane ring and the electron releasing group of two –OH groups on the catechol moiety at the ortho position^38^. The oxidation process of 5-CQA undergoes a reversible mechanism with transfer of two electrons and two protons^39^. Precisely, the formation of the anion 5-CQA^−^ by the pre-dissociation of a proton happens in the first step followed by the formation of radical by oxidation. Next, the radical releases a water molecule to form a carbocation which finally leads to the formation of quinone structure (as shown in Fig. 3). According to Jorge G. Uranga et al. pH is a crucial factor that depends the antioxidant activity of 5-CQA. As per their studies, it has been suggested that 5-CQA will forms o-quinone via a secondary oxidation reaction under basic conditions and at neutral conditions, it will be in the form of 5-CQA^–^. Generally, o-quinone possess a high antioxidant activity than anion formed by primary oxidation, so the reaction between 5-CQA and Ag^+^ occurs much faster at basic conditions than neutral^38^ (Fig. 3a). The mechanism of the formation of NPs consists of three steps; reduction, growth followed by termination process^40^. Initially Ag^0^ seeds are formed by the reduction of Ag^+^ by the 5-CQA^−^/ o-quinone, which results in small nuclei formation. These Ag^0^ seeds keep growing to form larger-sized NP aggregates with the characteristic of strong and intense plasmon band. During the termination process CAE–AgNPs are stabilized by COO^−^ groups of quinine derivatives, as well as resonance reaction of conjugate bonds present in 5-CQA derivatives in the vicinity of the surface via electrostatic interaction and a network of H-bonds, are formed in which acts as a protective cover around the CAE–AgNPs. Similar synthesis mechanism of metal NPs (Au and Ag) using gallic acid a naturally occurring polyphenol compound found in fruits, tea leaves, vegetables, etc. is already available in the literature^33^.

**Figure 5:**
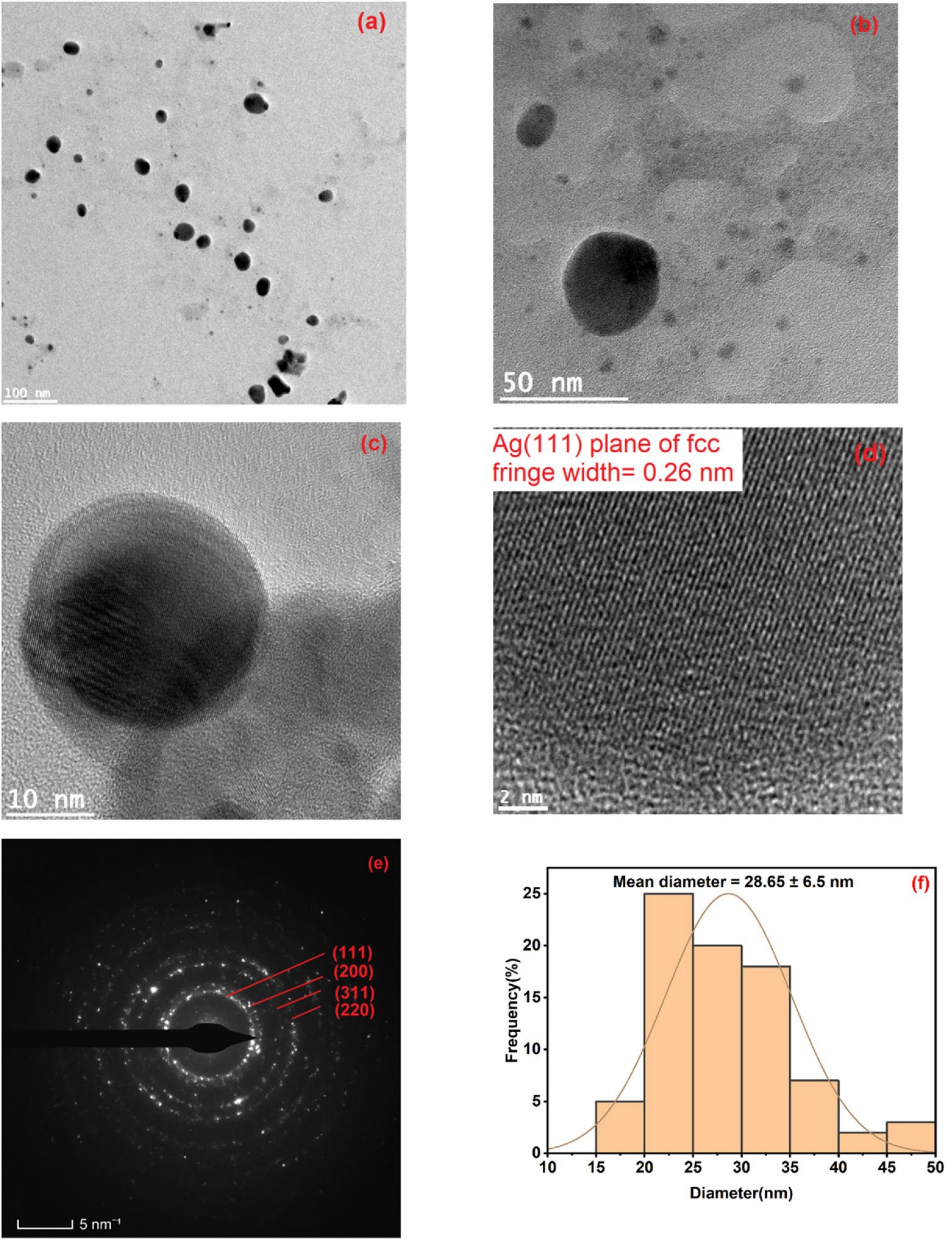
(**a**-**c**) TEM images of CAE-AgNPs at different magnifications. (**d**) TEM image of lattice fringe pattern. (**e**) SAED pattern. (**f**) Histogram for size distribution.

Morphological studies of CAE–AgNPs were investigated using TEM, showing that particles are dispersed well, and more or less spherical in shape (Fig. 5a–c). HRTEM images reveal the poly crystallinity of CAE–AgNPs with a clear lattice fringe with the spacing of 0.26 nm corresponding to Ag (111) plane of face-centred cubic (fcc) lattice^10^ (Fig. 5d). The selected area electron diffraction (SAED) pattern (Fig. 5e) confirms the (111), (200), (220) and (311) planes of AgNPs^18,41^. The average size of CAE–AgNPs estimated from TEM is 28.65 ± 6.5 nm (Fig. 5f) and which is found to be less than that obtained from DLS measurement (Fig. 2d) because there we are measuring the hydrodynamic diameter that gives us the information on the phytochemical cloud around the NPs.

### Surface characterization using FTIR & µRaman studies

**Figure 6:**
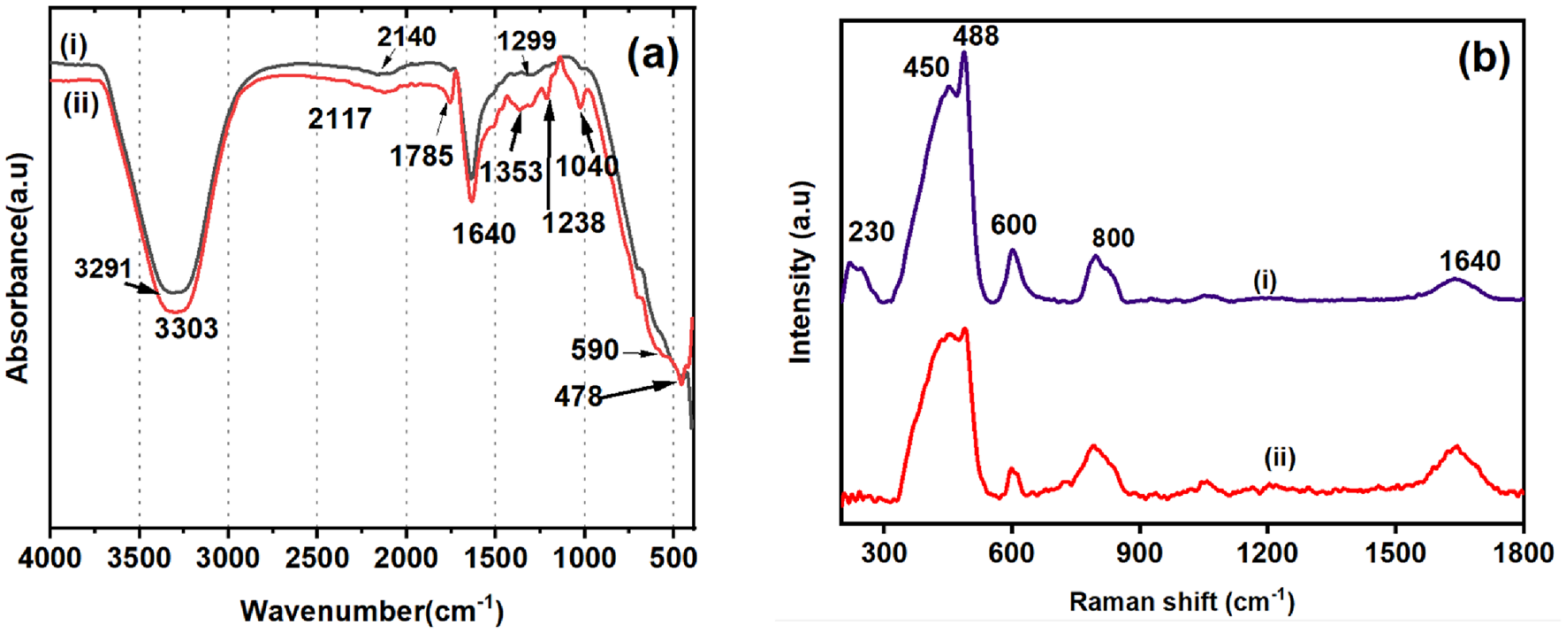
(**a**) FTIR spectra in the region 4000–400 cm^−1^ of (i) CAE (40 mg/ml) and (ii) CAE–AgNPs. (**b**) Raman spectra of (i) CAE (40 mg/ml) (ii) CAE–AgNPs in the region of 200–1800 cm^−1^.

FTIR a vibrational spectroscopic tool, is useful for the qualitative and quantitative analyses of materials due to its functional group specificity. FTIR studies of CAE and the synthesized CAE–AgNPs were carried out in water using ATR technique (Fig. 6a).

CAE exhibits characteristic bands (as tabulated in Table 1) at 3291, 2140, 1640 and 1299 cm^−1^. The reduction, capping and stabilization process causes some significant changes in the FTIR spectra of CAE–AgNPs like change in the spectral intensity and/or shift in the position of the peaks appear at 3306, 2117, 1640 and 1238 cm^−1^ compared to the CAE.

**Table 1:** FTIR vibrational mode assignment for CAE and CAE–AgNPs.

Sample	Wave number (cm^−1^)	IR assignments	Reference
CAE	1299	Vibrations of polyols	^42^
	1640	C = O stretching vibration	^43,42^
	2140	C–H vibrations	^44^
	3291	O–H stretch	^45,42^
CAE–AgNPs	478, 590	Ag–O, Ag–N vibrations	^45,42^
	1040, 1238	Vibrations of primary alcohol (–OH) and polyols	^43,42^
	1353	Aromatic band vibrations	^45^
	1640	C = O, C = N stretch	^44^
	3306	O–H stretch	^45,42^

Some additional peaks are also emerged in CAE–AgNPs and are situated at around 1785, 1353, 1238, 1040, 590 and 478 cm^−1^. These spectral assignments signify the potential role of phytochemicals present in CAE^13,42–46^ and interaction with AgNPs^43^. Here, the strong broadband centered at around 3291 cm^−1^ and weak band at around 2140 cm^−1^ are ascribed to the inter molecular hydrogen bonded network formed by the hydroxyl groups (–OH) of phenolic acids on the surface of the nanoparticle. The increased intensity of such bands in CAE–AgNPs compared to CAE can be due to the increased local concentration of the oxidized 5-CQA molecules to form such hydrogen bonding on the surface of AgNPs. Moreover, in the case of CAE–AgNPs, there is a slight shift is present on these bands (shifted to 3306 and 2117 cm^−1^), which reflects the crucial role of –OH groups in reducing Ag^+^ to AgNPs. The emergence and more pronounced absorbance value of the carbonyl band at 1785 cm^−1^ after the formation of CAE–AgNPs signifies interaction of the stabilizing carboxylate group of 5-CQA to the surface of the CAE–AgNPs and the existence of corresponding quinonic derivative as a result of oxidation of 5-CQA. The increased signal intensity of peak lies at 1640 cm^−1^ denotes the out-of-phase C = O stretching vibrations of 5-CQA and other phytochemicals presence in the vicinity of the surface of the CAE–AgNPs^43–45^.

The bands in the region 1300–1000 cm^−1^ are assigned to the presence of CGAs and these are attributed to the polysaccharides and other carbohydrates^43^. The stretching modes of Ag–O and Ag–C can be seen in the wavenumber regions 1353, 590 and 478 cm^−1^which confirms the reduction of Ag^+^ to AgNPs^47,48^. Hence, from the outcome of IR studies, it is proposed that CAE–AgNPs are stabilized via electrostatic interactions of carboxylate group of 5-CQA and its oxidized quinine form (Fig. 4). μRaman spectroscopy is a simple and reliable tool that provides a detailed fingerprint of molecules in a non-destructive and rapid manner. Figure 6b shows Raman spectra of CAE and CAE–AgNPs in the range of 200–1800 cm^−1^. The vibrational modes and their assignments related to the samples under study are presented in Table 2. For CAE, the major Raman vibrational modes are present at 450, 490, 600, 800 and 1640 cm^−1^. Similarly, for CAE–AgNPs, a new band appears at 230 cm^−1^ along with the other parent bands which show some shift in positions and variation in intensities. Ag–O bending vibrations^47,48^ are evident from the newly emerged band at 230 cm^−1^ in Fig. 6b. The increased intensity of the peaks after the formation of CAE–AgNPs located at 450, 488 and 600 cm^−1^ compared to CAE corresponds to out-of-plane bending vibrations of –OH and –C = O groups present in the phenolic contents of CAE and indicates the involvement of 5-CQA in the reduction mechanism of Ag^+^ ions^42,43,47^.

**Table 2:** Raman vibrational mode assignment for CAE and CAE–AgNPs.

Sample	Wavenumber (cm^−1^)	Raman assignments	Reference
CAE	450, 488	Out of plane bending vibrations of –OH	^43,42,47^
	600, 800	Out of plane bending vibrations of –C = O and –CH	^42,47^
	1640	C = O, C = N stretching vibrations	^43,42,47^
CAE–AgNPs	230, 450, 488	Ag–O, Ag–N vibrations Out of plane bending vibrations of –OH	^42,47,48^
	600, 800	Out of plane bending vibrations of –C = O and –CH	^42,47^
	1640	C = O, C = N stretching vibrations	^42,47^

Moreover, the vibrational assignments due to out-of-plane bending vibrations of –CH and stretching of C = O are also present at around the wavenumber regions 600 and 800 cm^−1^. These bands are characteristic bands of CGAs and caffeine present in CAE^42,47^. Hence, the role of 5-CQA and other bioactive elements present in CAE as reducing and capping agents during the formation of NPs is evident from the Raman spectral investigations of CAE and CAE–AgNPs, respectively.

### Selectivity and sensitivity studies of CAE–AgNPs

**Figure 7:**
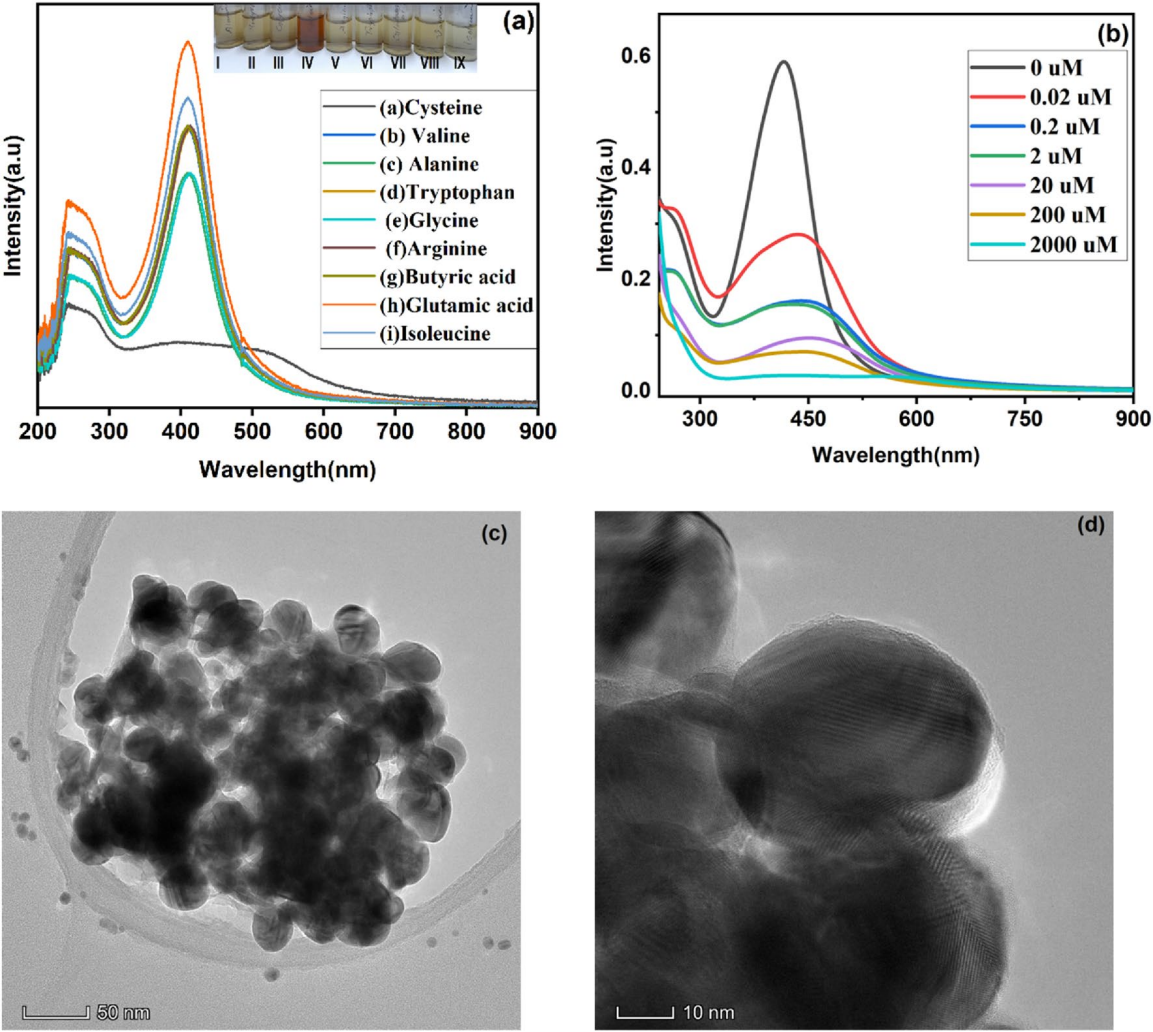
(**a**) UV-Vis spectra of selectivity study using different amino acids and the inset show color noticed for CAE-Ag colloid after the addition of nine amino acids (I- Ala, II- Ba, III- Gly, IV- Cys, V- Arg, VI-Trp, VII- Ga, VIII-Val and IX- Ils), respectively. (**b**) UV-Vis spectra of sensitivity study of L-Cys at different dilutions. (**c**) - (**d**) TEM images of CAE-AgNPs after adding L-Cys.

Interaction studies between polyphenols and amino acids are having a great concern in day-to-day life because it reveals the idea behind protein-phenol complex formation and mechanisms^49^. For example, Liang et al. reported the higher affinity of thiol group in cysteine for CGA o-quinones which was a complements knowledge of polyphenol-protein interaction that applied in sunflower protein food products which is recognized as one of top 6 plant-based food product^50^. On the other hand, the directing power in thiol-based redox mechanism of naturally occurring plant-based polyphenols and molecular mechanism involving phenol–protein interaction studies are unavoidable tools to battle over COVID-19 pandemic situation^21^. Hence, it is a relevant topic that must investigate about the interaction between prepared green synthesized AgNPs with thiol-contained amino acids. pKa value of 5-CQA is reported as 3.1; below this pH carboxylate group of 5-CQA is neutral in nature so that there will be no electrostatic interaction with CAE–AgNPs surface so that stabilization of CAE–AgNPs will not be take place^33^. The selectivity studies of nine different amino acids Ala, Ba, Gly, Cys, Arg, Trp, Gla, Val and Ils with CAE–AgNPs were carried out at neutal pH and corresponding colorimetric responses are noted (as shown in the inset of Fig. 7a).

From Fig. 7a, only the sample of AgNPs with L-Cys shows distinct color of dark reddish brown instead of pale yellow as in the case of others. The outcome indicates that synthesized CAE-AgNPs possess high selectivity towards the L-Cys sample than others. There are notably few spectral changes that occurred in the SPR absorption of AgNPs with the addition of other amino acids (Fig. 7a). In the case of L-Cys, the intensity of the parent plasmonic band of CAE-AgNPs drastically reduced and at the same time there is an appearance of a new peak at the higher wavelength around 560 nm. The shift in plasmonic band absorption is seemed to be directly proportional to concentration of L-Cys in the solution (as shown in Fig. 7b). It is mainly due to the chemical interaction between CAE–AgNPs and the analyte L-Cys which is followed by the aggregation of the particles that is confirmed from the TEM images shown in Fig. 7c,d. The corresponding size of the NPs was 48 ± 3.2 nm and from the former spherical shape, the morphology is modified into an oval. The increase in the average diameter is about 21.50 nm, indicating the aggregation of CAE–AgNPs. Further, an aqueous solution of L-Cys of various concentrations ranging from 0.02 to 2000 µM were used to test the sensitivity (Fig. 6b).

The intensity of the plasmon band of CAE–AgNPs centered at 414 nm decreases drastically and continually as the concentration of L-Cys is increased from 0.02 μM to 2000 μM. At the same time, there is an appearance of a new peak at the higher wavelength around 450 nm. The trend of decreasing intensity and appearance of a new peak at around 450 nm can be attributed to the affinity of CAE–AgNPs to make chemical bonds with L-Cys present as surface ligands which leads to the aggregation^51–53^. The aggregation induced plasmonic band shift is due to the coupling of plasmonic oscillations of corresponding metallic NPs which is widely utilized for colorimetric detection of analytes. Furthermore, higher the number of CAE–AgNPs and their proximity, higher will be the shift generated as a new resonance band. The complex formed with L–Cys and hydroxyl group of o-quinine form of 5-CQA on the surface of CAE–AgNPs bring those CAE–AgNPs closed to each other and results in the coupling of plasmonic oscillation and aggregation.

### Detection of L-Cys using CAE–AgNPs and its Mechanism

Being a low-cost colorimetric probe, green synthesized AgNPs are becoming a promising sensing tool among researchers. Mainly, their localized SPR (LSPR) property is utilized for the fabrication of LSPR-based nanosensors. The parameters like size, shape, inter-particle distance, the dielectric constant of the medium, surface characteristics and composition are in turn related to the LSPR phenomena. Here, the reason for distinct color change and drastically decreased absorption intensity of CAE–AgNPs after the addition of L-Cys (as shown in Fig. 7a–b) can be attributed to the adsorption of L-Cys on AgNPs surface via the plasmon-driven chemical transformations at the interface between AgNPs and L-Cys^42^. Hence, the approach of aggregation-based LSPR colorimetric sensing of CAE–AgNPs results in the reduction of inter-particle distance so that the adjacent particles start to overlap (Fig. 7c–d). Since AgNPs possess a high value of work function (4.26–4.75 eV), the binding of the NPs with electron-rich groups are much more favorable^54^. So, the increased number density of hotspots of CAE–AgNPs makes a transformation in the chemistry of such NPs via energy transfer and hot electron carriers and enhances the reaction between Ag and L-Cys for the formation of Ag–Cys metal–ligand complex^55^. The active organic functional sites (–COOH, –NH_3_ and –SH) of L-Cys act as ligating adsorbing species towards the CAE–AgNPs and allows to open a visual pathway for analyzing metal species with enhanced selectivity and sensitivity^7^. The aggregation induced by L-Cys in the colloidal solution of CAE–AgNPs can be explained by the chemical interaction between the surface of CAE–AgNPs and thiol group present in the L-Cys. The isoelectric point of L-Cys is 5.02 and the reaction of Cys with CAE–AgNPs is processing at a pH range of 6.3–6.7. So, the amino part of zwitterionic L-Cys will be neutralized and leaves an extra negative charge on the carboxyl tail (COO^−^)^7^. But compared to the carboxyl group, the greater affinity of the thiol group towards CAE–AgNPs induces a replacement of CAE in CAE–AgNPs and gets adsorbed on the surface of CAE–AgNPs through ligand exchange reaction.

**Figure 8:**
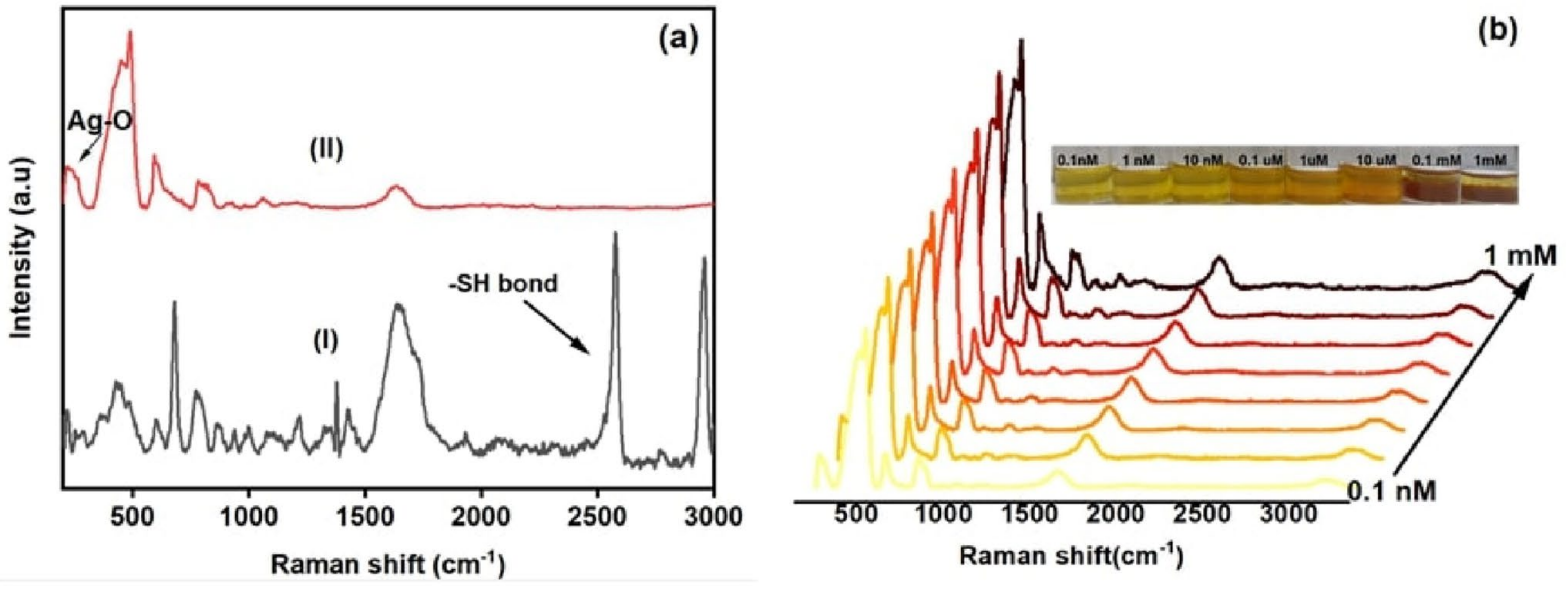
Raman spectra of (**a**) pure L-Cys (panel I) and CAE–AgNPs after the addition of L-Cys (panel II), (**b**) CAE–AgNPs after the addition of L-Cys at different concentrations (Inset shows the corresponding change in colour of samples) in the region 200–3000 cm^−1^.

Since FTIR–ATR miss the mark to identify the spectral signature of metal-S bonds below 400 cm^−1^, therefore we have followed the complimentary μRaman study where the acquired signals are susceptible to intermolecular interactions. Here, Fig. 8a (panel I–II) shows the μRaman spectra for pure L-Cys (panel I:black line) and CAE-AgNPs (panel II: red line) after the addition of L-Cys. Usually, L-Cys molecules form a disulfide bond (S–S) with adjacent L-Cys molecules and result in forming dimeric cystine form and results in the formation of the strong band around 488 cm^−1^.

Here, the increased intensity of Raman signal at 488 cm^−1^ in CAE–AgNPs after the addition of L-Cys (Fig. 8a) compared to CAE–AgNPs without L-Cys (Fig. 7b) can be attributed to the involvement of S–S bonding. On the other hand, the absence of the strong S–H stretching vibration of L-Cys at around 2550 cm^−1^ in CAE–AgNPs indicates the confirmation of binding of sulfur part from L-Cys to CAE–AgNPs surface after the cleavage of thiol S–H group^54,55^. Hence, Ag–S bindings are confirmed in the region 200–500 cm^−1^ and strengthen the possible evidence for the greater affinity of CAE–AgNPs towards sulphur ions. Additionally, the notable change in the shape of peak at around 800 cm^−1^ after the addition of L-Cys indicates the extent of bonding of L-Cys with AgNPs surface via carboxyl groups present in it^54,55^. Some previous theoretical findings suggested the fact that the amino group in L-Cys will be weakened due to the dominant nature of interconnecting H-bonds in the case of adsorption on the surface of CAE–AgNPs.

To find out the detection limit of our biosensor, a Raman study of different dilutions of aqueous L-Cys solution ranging from 0.1 nM to 1 mM using CAE–AgNPs (A1) as a substrate was performed (as depicted in Fig. 8b). However, Raman signals are observed even at a concentration of 0.1 nM, which indicates, that using our CAE–AgNPs as a substrate the lower detection limit for L-Cys can reach up to 0.1 nM, which is found to be better than previously reported Ag based L-Cys sensors (Table 3). Hence, it is ensured that our green synthesized CAE–AgNPs can be employed as an effective biosensor for rapid and direct detection of L-Cys, which can be applied to the biomedical and food industry.

**Table 3:** Comparison of detection limits of Ag-based L-Cys sensors with CAE–AgNPs.

Probe	Mechanism	LOD (nM)	Reference
Cellulose nanofibrils Capped AgNPs	Colourimetric	49	^7^
Baggase extract reduced AgNPs	Colourimetric	35	^30^
AgNPs in the presence of Cr^3+^	Colourimetric	1	^25^
Magnetite-Ag core shell nanocomposites	Inhibition based	87	^26^
Ag nanoprism dissolved in oxygen	Colourimetric	10	^27^
Dextran coated AgNPs	Optical change based	12 $\times {10}^{3}$	^28^
AgNPs-graphene quantum dots	Electrochemical	10	^24^
N-(1-Naphthyl)ethylene diamine cation stabilized AgNPs	Spectrophotometric	308	^29^
	Spectrofluorimetric	131	
CAE–AgNPs	Colourimetric	0.10	This work

## Conclusions

To the best of our knowledge, this work is the first attempt to demonstrate highly stable and ecologically friendly phytosynthesized AgNPs using CAE. The average diameter of the as-prepared CAE–AgNPs is 28.65 ± 6.5 nm having excellent stability with a zeta potential value around −57 mV even after 3 months from the synthesis. We have demonstrated the detailed mechanism which reveals the dominating reduction property of 5-CQA, the major polyphenol present in CAE. At neutral and basic pH conditions the oxidized forms 5-CQA^−^ and 5-CQA^2−^ of 5-CQA are actively participating in the reduction process via electrostatic interaction with electron rich COO and OH moieties and electron deficient Ag ions. As synthesized CAE–AgNPs were stabilized well via delocalization of conjugate electrons in the phenoxy ions and networking H-bonds. The stable colloidal suspension of CAE − AgNPs is further used for the colorimetric detection without aid of any sophisticated instrument facilities. The selective detection of L-Cys containing a thiol-group among 9 amino acids has been confirmed by monitoring the color change from yellow to brown–red with naked eyes and UV − Vis spectroscopy in just 5 min. Moreover, we have explored the detailed interaction of L-Cys on the surface of CAE–AgNPs using μRaman spectroscopy that showed a lower LOD even at 0.1 nM compared to the previously reported Ag-based L-Cys sensors. The excellent biosensing capability and remarkable antibacterial efficacy of CAE–AgNPs facilitate the applicability of the CAE–AgNPs towards biomedical areas with minimum toxicity effects. We believe, our study provides a new environmentally benign route for the synthesis of AgNPs in large-scale production.

## Data Availability

Data are available from the corresponding author on reasonable request.
